# Short-Term Changes in TNF-Alpha, IL-6 and Adiponectin Following Bariatric Surgery in Caucasian Obese Adults: An Observational Case–Control Study

**DOI:** 10.3390/medicina60111789

**Published:** 2024-11-01

**Authors:** Razvan-Marius Ion, Adina Hutanu, Daniela Tatiana Sala, Mircea Gabriel Muresan, Stefania R. Fodor, Septimiu Voidazan, Gabriela Beresescu, Radu Mircea Neagoe

**Affiliations:** 1 Doctoral School of Medicine and Pharmacy, “George Emil Palade” University of Medicine, Pharmacy, Sciences and Technology of Targu Mures, 540142 Targu Mures, Romania; razvan.ion@umfst.ro; 2 2nd Department of Surgery, Mures County Emergency Hospital, 540136 Targu Mures, Romania; tatiana.sala@umfst.ro (D.T.S.); radu.neagoe@umfst.ro (R.M.N.); 3 Center for Advanced Medical and Pharmaceutical Research, “George Emil Palade” University of Medicine, Pharmacy, Sciences and Technology of Targu Mures, 540136 Targu Mures, Romania; 4 Department of Laboratory Medicine, “George Emil Palade” University of Medicine, Pharmacy, Science and Technology of Targu Mures, 540142 Targu Mures, Romania; 5 Second Department of Surgery, “George Emil Palade” University of Medicine, Pharmacy, Science and Technology of Targu Mures, 540139 Targu Mures, Romania; 6 Anatomy Department, “George Emil Palade” University of Medicine, Pharmacy, Science and Technology of Targu Mures, 540139 Targu Mures, Romania; mircea.muresan@umfst.ro; 7 Department of Anestesiology, “George Emil Palade” University of Medicine, Pharmacy, Science and Technology of Targu Mures, 540109 Targu Mures, Romania; raluca.fodor@umfst.ro; 8 Anaesthesiology and Intesive Care Clinic, County Emergency Clinical Hospital of Targu Mures, 540136 Targu Mures, Romania; 9 Department of Epidemiology, “George Emil Palade” University of Medicine, Pharmacy, Science and Technology of Targu Mures, 540139 Targu Mures, Romania; septimiu.voidazan@umfst.ro; 10 Department of Morphology of Teeth and Dental Arches, Faculty of Dentistry, “George Emil Palade” University of Medicine, Pharmacy, Science and Technology of Targu-Mures, 540142 Targu-Mures, Romania; felicia.beresescu@umfst.ro

**Keywords:** severe obesity, LSG, interleukin-6, TNF-alpha, adiponectin

## Abstract

*Background and Objectives:* Overweight and obesity are well-known conditions that negatively impact the health and lifestyle of an individual. Bariatric surgery is one of the most efficient weight loss techniques. Besides the main effect on the bodyweight, improvement in the levels of inflammatory biomarkers, such as interleukin 6 (IL-6), tumor necrosis factor alpha (TNFalfa), and others, has been observed. The purpose of this study was to establish the correlations between obesity-linked chronic systemic inflammation (estimated with inflammatory cytokine levels) and the weight loss process after metabolic surgery. *Materials and Methods:* An observational cohort study included two categories: the patients with obesity–bariatric group and the patients without obesity–control group. The study was performed between 1 February 2021 and 1 March 2023. Baseline characteristics, anthropometrics, biochemical assessment and inflammatory biomarkers were measured both before surgery and one year after the procedure, in the case of the bariatric group. The control group was assessed in the same period as the pre-surgery bariatric group. The bariatric group underwent two types of bariatric procedures: the majority underwent laparoscopic sleeve gastrectomy whereas a select few underwent one anastomosis laparoscopic gastric bypass. *Results:* We performed a prospective study comprising 55 Caucasian patients—from which 33 patients had morbid obesity, a mean age of 41.76 ± 10.78 and a mean BMI of 43.34± 7.51 kg/m^2^. The preoperative levels of IL-6 were positively correlated with waist circumference (r = 0.354, *p* = 0.043), weight (r = 0.549, *p* = 0.001) and BMI (r = 0.520, *p* = 0.002). After applying the Kruskal–Wallis test and Dunn’s test, significant differences for IL-6 (*p* = 0.010) and adiponectin (*p* = 0.024) were obtained for values recorded pre- and post-surgery. No correlation was found between adiponectin, IL-6, TNF- α levels and anthropometric indices after surgery. Our study showed that bariatric surgery significantly changes the values of inflammatory cytokines one year after surgery. Nevertheless, we did not find significant correlations between the baseline values of these inflammatory markers and the weight loss process after surgery at a short-term (one-year) follow-up. *Conclusions:* Our study demonstrated that bariatric surgery significantly changes the level of inflammatory cytokines one year after operation. We demonstrate that preoperative levels of IL-6 are positively correlated with age, WC, and BMI.

## 1. Introduction

During the last few decades, overweight and obesity have become pandemic conditions; in the near future, the disease is expected to affect more than 4 billion people worldwide by 2035 [1]. Both conditions are considered individual risk factors for various health conditions, including hypertension, cardiovascular disease, diabetes, steatohepatitis and even certain types of cancer [2]. The pathogenic pathways of obesity are multiple and incompletely known; a modified diet, lack of physical activity, stress, genetic and metabolic disturbances are all involved in the process [3].

Many of the obesity related negative health consequences are associated with inflammation and its consequences [3]. Adipocytes exert an influence on several physiological processes, including pancreatic beta-cell function, hepatic glucose production, muscle glucose assimilation, appetite adjustment, and vascular inflammation [3,4]. This effect is mediated by a variety of adipocytokines, such as adiponectin, leptin, resistin, tumor necrosis factor-alpha (TNF-α), interleukins and other biomolecules that regulate the ration between appetite, food intake and body weight [4,5]. The source of some pro-inflammatory cytokines, such as tumor necrosis factor alpha (TNF-α) and interleukin-6 (IL-6), may be identified in the abdomen, where the white adipose tissue (WAT) is predominantly located and defined as visceral adipose tissue [5]. These cytokines are considered reliable indicators of inflammation [4,5].

Bariatric surgery is a very effective therapeutic approach for individuals with morbid obesity, leading to significant weight loss and relief of associated metabolic disorders [6]. The link between metabolic surgery and obesity-related chronic inflammation is repeatedly described in the literature but the results are debatable. A systematic review indicates a significant reduction in the levels of several inflammatory cytokines, including CRP, IL-6 and TNF-α, the most commonly measured inflammatory factors in bariatric surgery research [4]. A recent study showed that lower levels of HDL cholesterol (HDLc) are associated with a reduced likelihood of T2DM remission after surgery. Similarly, elevated levels of hsCRP are linked to a low insulin secretory reserve, which decreases the chances of post-surgical T2DM remission [6]. Furthermore, the relationship with other variables, such as weight loss, blood pressure reduction, and changes in lipid levels, varies inconsistently across bariatric surgery studies [4,5,6].

In a previous narrative review, we described the link between chronic inflammation, obesity and metabolic syndrome, highlighting the role of weight loss, particularly after bariatric procedures, in reducing obesity-related pro-inflammatory status and comorbidities [7]. The goal of this study is to establish the possible correlations between obesity-linked chronic systemic inflammation (such as that in diabetes, cardiovascular diseases, and atherosclerosis), evaluated through the cytokine plasma profile and weight loss process after metabolic surgery.

## 2. Materials and Methods

### 2.1. Patients

Our observational cohort study included two categories of patients: those with obesity (bariatric groups) who were admitted for bariatric surgery between 1 February 2021 and 1 March 2023 to two healthcare facilities from Targu Mures (Romania) and patients without obesity, admitted for the surgical treatment of other diseases (control group). All bariatric surgeries were performed by a single team and the inclusion criteria respected the 1991 National Institute of Health Bariatric Surgery Guidelines: patients with body mass index (BMI) between 35 and 39.9 kg/m^2^, with at least one of the main obesity-related diseases, i.e., type 2 diabetes mellitus (T2DM), arterial hypertension, obstructive sleep apnea syndrome (OSAS), or metabolic syndrome and patients with morbid obesity with BMI above 40 kg/m^2^. In fact, our bariatric group included only patients with morbid obesity. The non-bariatric procedures were performed by other senior surgeons from the public department. Initially, 33 patients were enrolled in each group, although from the control group, 10 patients were excluded due to complications during surgery or COVID-19 infection. In this context, in the control group, only 22 subjects were further considered in the study.

Exclusion criteria were generally those specific to bariatric or general surgery and general anesthesia: severe systemic diseases, serious blood and autoimmune disorders, severe mental health problems, and terminal diseases. We also excluded patients with overweight and grade 1 obesity and those with active or chronic diseases notably linked to inflammation (Crohn’s disease, chronic pancreatitis), patients taking anti-inflammatory, antibiotic, antiviral medications or under other chronic drug therapies, patients with active alcohol and/or illicit drug abuse and those with incomplete data in their medical records. Our study started when the country was still under restrictions imposed by COVID-19 infection; therefore, we also excluded these patients (all cases who underwent surgery were tested for COVID-19 infection).

All patients were informed about the aim of the study and the procedures to follow, and consented accordingly. The study was approved by both Hospital’s Ethics Committees: Emergency Mures County Hospital (protocol code 3570, on 19 February 2021) and Targu Mures Private Hospital Topmed (registration number 114, on 16 March 2021), and was conducted in accordance with the principles set forth in the Helsinki Declaration.

The primary endpoint of this study was to establish the early impact of bariatric surgery on adipokines (mainly adiponectin) and inflammatory cytokines (TNF-alpha and IL-6) in the group of patients with obesity. The secondary endpoint was to find out if these inflammatory cytokines measured at baseline have a predictive role for weight loss after bariatric surgery at a short-term follow-up. In our view, inflammatory cytokines are markers that change after surgery, and we considered it important to identify their correlation with bariatric surgery. We used a group of patients without obesity as a control, in order to establish the extent of the inflammatory response on the patients before the surgical procedures.

### 2.2. Clinical Assessments

Preoperative assessment of the patients was performed by a multidisciplinary team, and consisted of medical history evaluation, physical examination and relevant laboratory tests. Anthropometric measurements were performed before the surgery and included body height (BH) to the nearest 0.5 cm, body weight (BW) to the nearest 0.1 kg and waist circumference (WC), measured midline between the anterior superior iliac crest and the lowest rib. Body mass index (BMI) was calculated by dividing weight (expressed in kilograms) by squared height (measured in meters). Blood pressure and heart rate were measured using a semiautomated oscilometric device following standard recommendations. Relevant clinical data (on comorbidities such as hypertension, diabetes mellitus, dyslipidemia) and laboratory data from routine patient evaluations (lipid profiles) were also recorded.

### 2.3. Biochemical Assessments and Inflammatory Biomarkers

Blood samples were obtained through vacuum venipuncture after at least of 8 h fasting. For cytokine measurement, the blood samples were collected in serum separator tubes (SST), before and one year after surgery. The tubes were sent to the laboratory, and centrifuged for 10 min at 1000× *g* and 4 °C. Serum samples were retrieved in cryovials and stored at −80 °C until all patients were evaluated and analyzed at the end of the recruitment. The serum levels of cytokines of interest were evaluated using ELISA protocols on automated equipment, Dynex DSX ELISA (Dynex Technology, Chantilly, VA, USA), in the laboratory of the Center for Advanced Medical and Pharmaceutical Research of UMPhST of Targu Mures, Romania. According to the manufacturer’s recommendations, all three cytokines (IL-6, TNFalpha, and adiponectin) were quantified using a sandwich ELISA protocol with commercially available ELISA kits (Demeditec Diagnostics GmbH, Kiel, Germany). According to the manufacturer, the performance of the tests was at the detection limit of 0.7 pg/mL for TNF-alfa, 2 pg/mL for IL-6, and 0.59 ng/mL for adiponectin. The coefficients of variation for intra-assay and inter-assay precision were less than 10% for all parameters.

### 2.4. Surgical Intervention Protocol

Patients with obesity underwent 2 types of bariatric procedures: the majority were submitted to laparoscopic sleeve gastrectomy (LSG), the steps of the procedure being previously described [8] with no major technical differences. A small number of patients from this group (i.e., bariatric group) underwent one anastomosis laparoscopic gastric bypass (OAGB), when a gastrojejunostomy was performed 180 cm below the duodenojejunal angle in a side-to-side antecolic manner, with a linear stapler or manually. The intersurgeries performed in the control group were partial or total thyroidectomy, laparoscopic cholecystectomy, and transabdominal pre-peritoneal or totally extraperitoneal inguinal hernia repair.

### 2.5. Follow-Up

All our patients were followed up prospectively at a 3-month period in the first year after surgery. The data were collected during postoperative visits; we noted the weight loss process, the influence of the bariatric procedure on the main obesity-associated comorbidities, and other clinical and technical aspects related to the procedure. Blood samples were collected for all patients at one year follow-up for further examination of biochemical, metabolic, and inflammatory profiles.

### 2.6. Statistical Analysis

SPSS 23.0 software was used for statistical analysis of the data. Quantitative variables were tested for normality of distribution using the Kolmogorov–Smirnov test and were characterized by median and minimum–maximum or by mean and standard deviation (SD), when appropriate. Multivariate analysis was carried out using linear regressions. We used as a dependent variable the delta BMI. We included as independent variables TNF-alpha, IL-6, adiponectin and parameters of lipid profile. Student’s test and ANOVA (as well as the Bonferroni test for multiple comparisons) were applied for quantitative variables, which were expressed as mean and standard deviation. For variables that did not have a Gaussian distribution, the data were expressed as the median by applying the Mann–Whitney/Kruskal–Wallis test and Dunn’s test for multiple comparisons. The correlations between anthropometric measurements and laboratory values (lipid profile and inflammatory cytokines) were tested using Pearson and Spearman correlation coefficient. Statistical significance was considered at a *p*-value ≤ 0.05.

## 3. Results

During the above-mentioned period, 55 Caucasian patients were considered eligible for this study. We had 33 patients with morbid obesity (5 males, 15.1%), a mean age of 41.76 ± 10.78 and a mean BMI of 43.34 ± 7.51 kg/m^2^; in this “obesity group”, we had 7 patients with super-obesity, with a BMI exceeding 50 kg/m^2^. In the control group, we included 22 lean patients (9 males, 40.9%) with a mean age of 55.5 ± 14.23 and a mean BMI of 24.02 ± 1.71 kg/m^2^. In the obesity group, we had 31 patients who underwent an LSG and 2 patients who were submitted to a OAGB. The main demographic, anthropometric and laboratory data of our cohort are comparatively shown in Table 1, before and one year after surgery for the bariatric group (BG 0 and BG 1) and for the control group, respectively (CG) (Table 1 insertion). We had statistically significant changes in anthropometric parameters before and one year after the operation, i.e., bodyweight, waist circumference, and BMI. Even if it did not reach statistical significance, the lipid profile showed decreased LDL and TG and higher HDL values one year after bariatric surgery compared with BG 0 and CG.

**Figure 1:**
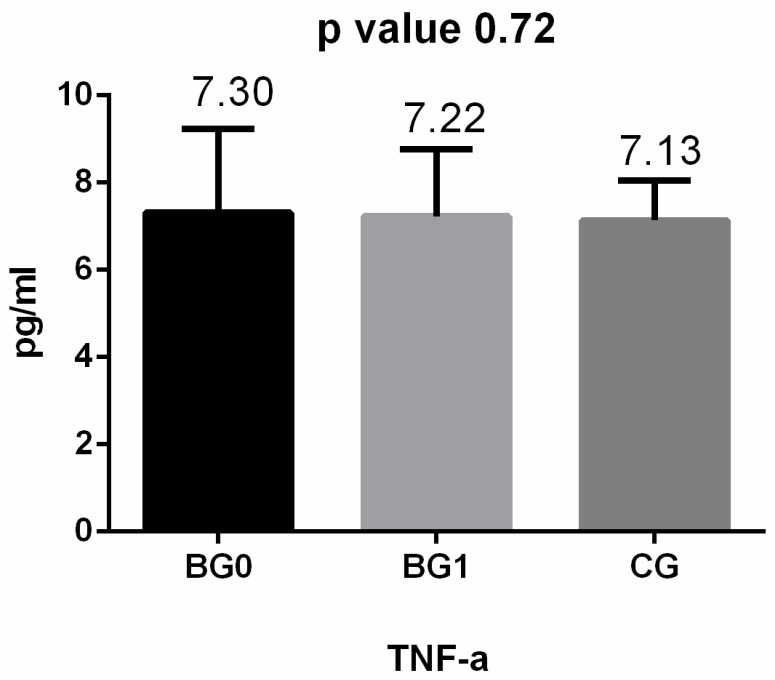
TNF-alpha levels in the studied groups: BG 0—before the bariatric procedure; BG 1—one year after the bariatric procedure; CG—control group.

**Figure 2:**
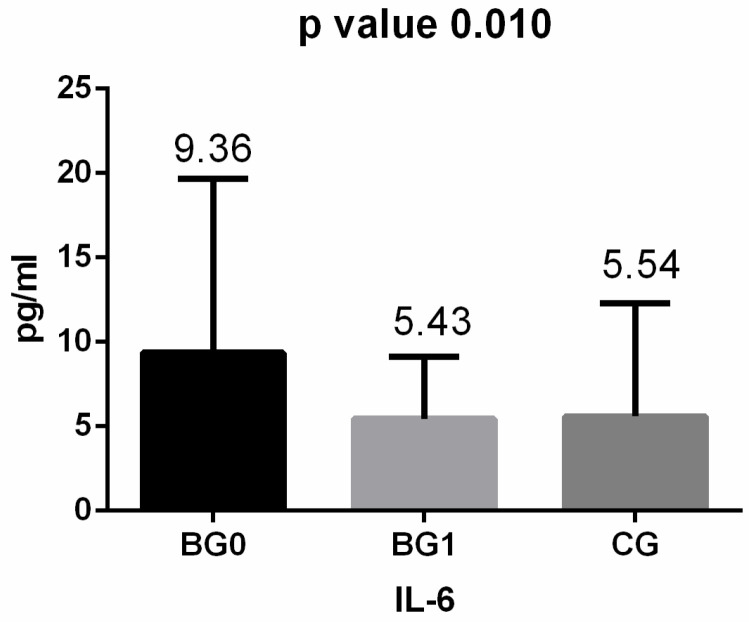
IL-6 levels in the studied groups: BG 0—before the bariatric procedure; BG 1—one year after the bariatric procedure; CG—control group.

**Figure 3:**
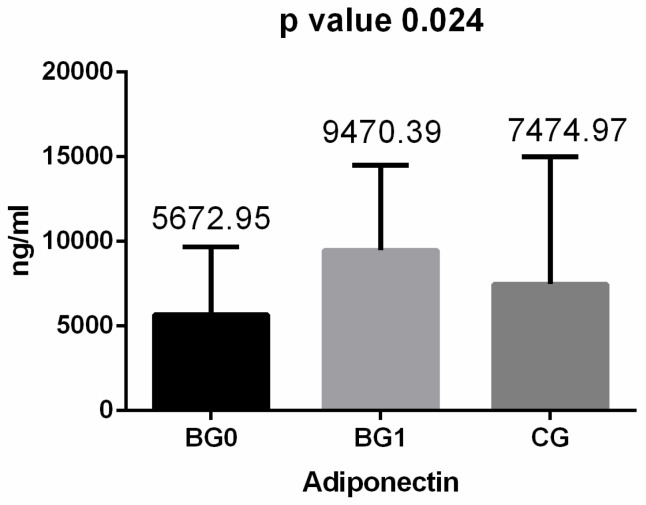
Adiponectin levels in the studied groups: BG 0—before the bariatric procedure; BG 1—one year after the bariatric procedure; CG—control group.

Regarding the inflammatory cytokines, we observed that after surgery, adiponectin values increased considerably (almost doubled), while values for IL-6 and TNF-α decreased (Figure 1, Figure 2 and Figure 3). After applying the Kruskal–Wallis test and Dunn’s test of multiple comparisons for the whole study group, significant differences for IL-6 (*p* = 0.010, *p* < 0.05) and adiponectin (*p* = 0.024, *p* < 0.05) were obtained; for IL 6, the median value in the BG 0 group was 9.36 pg/mL, significantly higher compared with that for BG 1 and CG (*p* = 0.024).

The correlations between anthropometric indices and lipid profile before surgery are detailed in Table 2. There was a positive correlation, before the surgery (BG 0), between total cholesterol values and both waist circumference (r = 0.378, *p* = 0.030) and body mass index (r = 0.389, *p* = 0.025). For pro-inflammatory cytokines, only IL-6 showed a positive correlation with BMI (r = 0.520, *p* = 0.002) and WC (r = 0.354, *p* = 0.043).

One year later (BG 1 group), changes in cholesterol levels were positively correlated with age (r = 0.409, *p* = 0.018) and waist circumference (r = 0.351, *p* = 0.045). LDL-C levels were also positively correlated with age (r = 0.369, *p* = 0.035) and waist circumference (r = 0.375, *p* = 0.031). However, for HDLc and TG, no correlations were found after surgery with any of these anthropometric measurements.

Table 3 shows the correlations between anthropometric indices and inflammatory cytokines before and after surgery. We note that preoperative levels of IL-6 have a positive correlation with BMI (r = 0.520, *p* = 0.002) and WC (r = 0.354, *p* = 0.043) but no correlations with lipid profile parameters (*p* > 0.05); for the moment after surgery, no correlation was found with the above-mentioned parameters. No correlation was found between adiponectin or TNF alfa and anthropometric or lipid metabolic parameters, at both moments (*p* > 0.05).

The multivariate regression model, where the dependent variable was Delta BMI, and the independent variables were inflammatory markers and lipid parameters, shows no significant associations (Table 4 and Table 5). This Delta BMI was obtained by the differences between BG 0 and BG 1, which were expressed by the mean ± standard deviation (16.42 ± 5.66).

## 4. Discussion

Our study showed that bariatric surgery significantly changes the values of IL-6, a potent pro-inflammatory cytokine, and adiponectin one year after surgery. Nevertheless, we did not find significant correlations between baseline values of these inflammatory markers and the weight loss process after surgery at a short-term (one-year) follow-up.

A huge body of evidence indicates bariatric surgery as an established method for promoting weight loss, its results exceeding those of other non-surgical methods [9]. Surgical management of obesity also has other benefits in addition to weight loss, such as improving blood pressure, correcting diabetes, sleep apnea, and dyslipidemia [9,10]. The mechanisms involved are complex and incompletely known, and show noticeable differences between the types of bariatric procedures [2,7,9]. Among these pathways, the role of inflammation has been extensively studied in recent years. In this view, it is already well known that bariatric surgery induces a significant reduction in adipose tissue mass and, consequently, an important decrease in adipocytokine plasma levels [7]. Even though the above-mentioned relation is well acknowledged, there are still inconsistencies in different studies relating to the pre- and postoperative values of circulating cytokines [7,9].

Inflammation is a useful tool used by the body to defend itself and repair damaged tissues [11]. However, a long-standing low-grade inflammatory status might be dangerous since it could prelude different diseases, i.e., atherosclerosis, diabetes, and some types of cancers [12,13,14]. The link between chronic low-grade systemic inflammation and obesity is well documented, with several pathogenic pathways already described; among them, the immune activation of excessive white adipose tissue (WAT), predominantly located intra-abdominally, is of paramount importance [3]. Visceral adipose tissue may be looked at as an active endocrine organ, responsible for releasing an important number of cytokines in plasma [5]. In obesity, due to increased body mass, the adipose tissue is dysfunctional, with an altered expression of cytokines and adipokines; as a result, pro-inflammatory plasma levels of cytokines such as interleukin-6 and tumor necrosis factor alpha increase in obesity patients [15]. Both markers are considered the most important pro-inflammatory cytokines because they have good sensitivity and sensibility in systemic chronic inflammation signaling, despite being less available due to higher costs [16]. Adiponectin plasma, on the other hand, is decreased in obese patients, and a level of it is correlated with increased risk for cardiometabolic diseases and insulin resistance [7,16]. There are pieces of evidence that changes in anthropometric indices, i.e., BMI, body fat, and waist circumference, are negatively correlated with adiponectin plasma level after weight loss due to the Mediterranean diet, exercise program or bariatric surgery [17]. In our study, we observed a significant increase in adiponectin level one year after metabolic surgery, our results being in line with those reported by the majority of authors [4]. However, when analyzing the influence of bariatric surgery on the plasma levels of these cytokines, the literature still offers debatable results [7,9]. In a systematic review and meta-analysis of studies published in 2019, when investigating IL-6 as an outcome measure after bariatric surgery, Askarpour et al. noted a relatively constant inverse influence of metabolic surgery on IL-6 plasma levels at 6 months or more of follow-up, with the results being dependent on the type of bariatric procedure and initial BMI. The same effect was noted on TNF-α levels at a one-year follow-up, but the results were not linked with the type of metabolic surgery [4]. In spite of this, some studies contradict, at least partially, these results; Rao SR, in a meta-analysis that included 48 studies, found significant differences only for IL-6 and C reactive protein (CRP) but not for TNF- α [18], the same results also being reported by others [19]. There are also differences when the temporality of metabolic and inflammatory changes after bariatric surgery are studied; in a recent paper, Villareal-Calderon et al. stated that metabolic shifts appear earlier after bariatric metabolic surgery, these being a requisite for inflammatory changes [20]. In our study we noted a significant reduction in both pro-inflammatory cytokines i.e., IL-6 and THF-α, and an increased level of adiponectin at a one-year follow-up, but with no correlation with anthropometric indices after surgery.

A considerable subpopulation of morbid obesity patients have dyslipidemia with elevated total cholesterol, LDL cholesterol and triglyceride, and low HDL cholesterol. We also studied lipid profile in our patients but our findings showed some inconsistencies. Inflammation and immune function are significantly regulated by lipid species and derivatives, including high-density lipoproteins (HDLs), which possess immunomodulatory and anti-inflammatory properties due to their ability to carry bioactive lipids and anti-oxidant proteins. At a mean BMI of 43 kg/m^2^, our study population (bariatric group) had “moderate” baseline dyslipidemia; we noted a correlation between baseline and one-year follow-up cholesterol values and waist circumference (which is a recognized predictor of visceral fat) but these data were not supported by the levels of other cholesterol fractions i.e., LDL and HDL cholesterol, and triglyceride. Even though we did not reach statistical significance, our results were in line with those already reported: metabolic surgery has a positive effect on lipid profile, lowering triglyceride, total cholesterol and LDL cholesterol, and increasing the level of HDL cholesterol [21,22].

The study of Liu and collab. found that adolescents with a ΔBMI above the threshold value, despite not being overweight at baseline, could develop significant metabolic abnormalities as adults. Delta BMI can be used as a predictor of MetS in early adulthood. Adolescents with a significant Delta BMI revealed the greatest risk of developing metabolic syndrome (MetS) [23]. In our study, we did not find significant associations of Delta BM with inflammatory levels and lipid parameters.

We identified some limitations of our study; it had a small number of patients for both bariatric and non-bariatric arms, so the statistical power might have been affected. We also failed to “match” the case with the control group due to significant differences noted in demographic characteristics within the groups (age, sex). The follow-up period was short, which might have also influenced the results. Selection bias was introduced when we included the small number of patients who underwent a bypass procedure. We did not include in our analysis other recognized inflammatory markers, such as C reactive protein, which might have given us different results. It was also a two-center analysis; therefore, our results cannot be extrapolated to a large-scale population.

## 5. Conclusions

Our study demonstrated that bariatric surgery significantly changes the level of inflammatory cytokines one year after operation. We demonstrate that preoperative levels of IL-6 are positively correlated with WC and BMI. This study showed that bariatric surgery influences the lipid panel in our study group but did not reach statistical significance. More powerful studies are needed to confirm or infirm our results.

## Figures and Tables

**Table 1:** Demographic, anthropometric and laboratory data in the study groups: BG 0—before the bariatric procedure; BG 1—one year after the bariatric procedure; CG—control group.

Variable	BG 0 (*n* = 33)	BG 1(*n* = 33)	CG(*n* = 22)
Age (years)	41.76 ± 10.78 **	41.76 ± 10.78	55.55 ± 14.23 ^££^
WC (cm)	133.73 ± 22.30 **	96.30 ± 12.17 ^##^	84.09 ± 9.04 ^£^
Height (cm)	166.21 ± 6.37	166.42 ± 6.27	169.77 ± 7.43
Weight (kg)	128.27 ± 23.04 **	82.85 ± 12.87 ^##^	69.50 ± 8.93 ^£^
BMI	43.34 ± 7.51 **	29.91 ± 4.41 ^##^	24.02 ± 1.71 ^££^
Cholesterol (total) (mg/dL)	181.63 ± 49.48	194.00 ± 55.98	175.84 ± 30.59
LDL-C (mg/dL)	121.53 ± 38.21	115.73 ± 40.67	113.27 ± 25.87
HDL-C (mg/dL)	47.79 ± 13.15	53.82 ± 12.16	52.94 ± 6.35
TG (mg/dL)	150.13 ± 67.80 **	124.54 ± 45.26	106.07 ± 26.75
Glucose (mg/dL)	111.11 ± 31.72	100.12 ± 19.39	99.5 ± 14.84
Creatinine (mg/dL)	0.86 ± 0.14	0.96 ± 0.17	0.76 ± 0.15
HTC(%)	41.61 ± 3.37	40.66 ± 4.11	39.96 ± 7.84
Hgb (g/dL)	13.71 ± 1.28	13.72 ± 1.48	15.1 ± 5.84
GOT (UI/I)	* 24.22 (12.1–212.87)	* 20 (10–128)	* 19.9 (12–35.8)
GPT (UI/I)	* 25.36 (12.65–248.4)	* 19 (5–99)	* 20.5 (8.7–47.6)
GGT (U/I)	* 28.93(13–66)	* 22 (13–46)	* 20 (7–126)
TNF alpha (pg/mL)	* 7.3 (4.74–89.56)	* 7.22 (4.91–10.58)	* 7.13 (4.34–11.13)
IL-6 (pg/mL)	* 9.36 (2.9–98.03)	* 5.43 (1.27–45.13)	* 5.59 (1.78–56.02)
Adiponectin (ng/mL)	* 5672.95 (944.24–14,120.51)	* 9470.39 (2.35–26,116.26)	* 7474.97 (2341.2–19,834.39)

WC, waist circumference; BMI, body mass index; LDL-C, low-density-lipoprotein cholesterol; HDL-C, high-density-lipoprotein cholesterol; TG, total triglycerides; HTC, hematocrit; Hgb, hemoglobin; GOT, aspartate aminotransferase; GPT, alanine aminotransferase; GGT, gamma glutamyl transferase; TNF-alpha, tumor necrosis factor-alpha. Data are expressed by the mean ± SD (standard deviation). The differences between groups were analyzed by ANOVA and a Bonferroni test for multiple comparisons. * Data were expressed by the median (minimum and maximum). The differences between groups were analyzed by a Kruskal–Wallis test and Dunn’s test for multiple comparisons. CG compared to BG 0: * *p * <  0.05; ** *p*  <  0.01. BG 0 compared to BG 1: ^##^ *p* < 0.01. BG 1 compared to CG: ^£^ *p* < 0.05; ^££^ *p* < 0.01.

**Table 2:** Correlations between cholesterol total, LDL-C, HDL-C and triglyceride and anthropometric indices (age, BMI, WC) before (BG 0) and after bariatric surgery (BG 1).

		Age		BMI		WC	
		BG 0	BG 1	BG 0	BG 1	BG 0	BG 1
Cholesterol (total) (mg/dL)	*p*-value Pearson correlation	0.719 −0.065	0.018 0.409	0.030 0.378	0.273 0.197	*0.025* *0.389*	0.045 0.351
ldl-C (mg/dL)	*p*-value Pearson correlation	0.859 −0.032	0.035 0.369	0.254 0.204	0.144 0.260	0.642 0.084	0.031 0.375
hdl-C (mg/dL)	*p*-value Pearson correlation	0.745 0.059	0.149 0.257	0.747 −0.058	0.781 −0.050	0.371 0.161	0.977 0.005
tg (mg/dL)	*p*-value Pearson correlation	0.460 0.133	0.936 −0.014	0.572 0.102	0.093 0.298	0.109 0.284	0.134 0.266

WC, waist circumference; BMI, body mass index; LDL-C, low-density-lipoprotein cholesterol; HDL-C, high-density-lipoprotein cholesterol; TG, total triglycerides.

**Table 3:** Correlation between adiponectin, IL-6, TNF-α, laboratory parameters and anthropometric markers before (BG 0) and after (BG 1) surgery (determined by Spearman’s correlation coefficient).

		Adiponectin		IL-6		TNF-α	
		BG 0	BG 1	BG 0	BG 1	BG 0	BG 1
Age (years)	*p*-value Spearman’s rho	0.221 0.219	0.465 0.132	0.943 0.013	0.913 −0.020	0.978 0.005	0.524 −0.115
WC (CM)	*p*-value Spearman’s rho	0.076 −0.313	0.193 −0.233	0.043 0.354	0.229 0.215	0.168 0.246	0.909 −0.021
BMI	*p*-value Spearman’s rho	0.146 −0.258	0.899 −0.023	0.002 0.520	0.273 0.197	0.140 0.262	0.722 −0.064
Cholesterol (mg/dL)	*p*-value Spearman’s rho	0.948 −0.012	0.698 0.070	0.213 0.222	0.633 0.086	0.615 0.091	0.417 −0.146
ldl-C (mg/dL)	*p*-value Spearman’s rho	0.410 0.148	0.600 0.095	0.657 −0.080	0.842 0.036	0.965 −0.008	0.965 0.008
hdl-C(mg/dL)	*p*-value Spearman’s rho	0.864 0.031	0.102 0.209	0.671 −0.077	0.734 0.061	0.647 0.083	0.698 −0.070
tg(mg/dL)	*p*-value Spearman’s rho	0.109 −0.284	0.787 0.049	0.827 0.040	0.583 0.099	0.086 0.304	0.800 0.046

WC, waist circumference; BMI, body mass index; LDL-C, low-density-lipoprotein cholesterol; HDL-C, high-density-lipoprotein cholesterol; TG, total triglycerides; HTC, hematocrit; HGB, hemoglobin; GOT, aspartate aminotransferase; GPT, alanine aminotransferase; GGT, gamma glutamyl transferase.

**Table 4:** The multivariate regression model including the Delta BMI of the change in inflammatory parameters.

Independent Variables	Coefficient	Std. Error	t	*p*
TNF-ALPHA	0.00001466	0.00009814	0.149	0.8823
IL-6	0.00007882	0.00006362	1.239	0.2253
ADIPONECTIN	0.0000001455	0.0000001507	0.965	0.3424

**Table 5:** The multivariate regression model including the Delta BMI of the change in lipid profile values.

Independent Variables	Coefficient	Std. Error	t	*p*
COLESTEROL-TOTAL (mg/dL)	0.02335	0.01897	1.231	0.2287
HDL-C (mg/dL)	0.1358	0.06961	1.950	0.0612
LDL-C (mg/dL)	−0.005956	0.02346	−0.254	0.8014
TG (mg/dL)	0.009631	0.01331	0.724	0.4753

## Data Availability

The datasets used and analyzed during the current study are available from the corresponding author on reasonable request.
